# Enhancing the Activation and Releasing the Brakes: A Double Hit Strategy to Improve NK Cell Cytotoxicity Against Multiple Myeloma

**DOI:** 10.3389/fimmu.2018.02743

**Published:** 2018-11-27

**Authors:** Sara Tognarelli, Sebastian Wirsching, Ivana von Metzler, Bushra Rais, Benedikt Jacobs, Hubert Serve, Peter Bader, Evelyn Ullrich

**Affiliations:** ^1^Childrens Hospital, Experimental Immunology, Johann Wolfgang Goethe University, Frankfurt, Germany; ^2^Childrens Hospital, Department of Pediatric Stem Cell Transplantation and Immunology, Johann Wolfgang Goethe University, Frankfurt, Germany; ^3^LOEWE Center for Cell and Gene Therapy, Johann Wolfgang Goethe University, Frankfurt, Germany; ^4^Department of Hematology and Oncology, Johann Wolfgang Goethe University, Frankfurt, Germany; ^5^Department of Haematology and Oncology, University Hospital Erlangen, Erlangen, Germany; ^6^German Cancer Consortium (DKTK), Heidelberg, Germany; ^7^German Cancer Research Center (DKFZ), Heidelberg, Germany

**Keywords:** multiple myeloma, autologous stem cell transplantation, NK cells, adoptive cell therapy, NKG2A blocking, checkpoint inhibition

## Abstract

Natural killer (NK) cells are innate lymphocytes with a strong antitumor ability. In tumor patients, such as multiple myeloma (MM) patients, an elevated number of NK cells after stem cell transplantation (SCT) has been reported to be correlated with a higher overall survival rate. With the aim of improving NK cell use for adoptive cell therapy, we also addressed the cytotoxicity of patient-derived, cytokine-stimulated NK cells against MM cells at specific time points: at diagnosis and before and after autologous stem cell transplantation. Remarkably, after cytokine stimulation, the patients' NK cells did not significantly differ from those of healthy donors. In a small cohort of MM patients, we were able to isolate autologous tumor cells, and we could demonstrate that IL-2/15 stimulated autologous NK cells were able to significantly improve their killing capacity of autologous tumor cells. With the aim to further improve the NK cell killing capacity against MM cells, we investigated the potential use of NK specific check point inhibitors with focus on NKG2A because this inhibitory NK cell receptor was upregulated following *ex vivo* cytokine stimulation and MM cells showed HLA-E expression that could even be increased by exposure to IFN-γ. Importantly, blocking of NKG2A resulted in a significant increase in the NK cell-mediated lysis of different MM target cells. Finally, these results let suggest that combining cytokine induced NK cell activation and the specific check point inhibition of the NKG2A-mediated pathways can be an effective strategy to optimize NK cell therapeutic approaches for treatment of multiple myeloma.

## Introduction

Multiple myeloma (MM) is a malignancy of terminally differentiated plasma cells (PCs). The hallmarks of the disease are an excess of monoclonal PCs in combination with monoclonal protein in the blood and/or urine (1). Standard therapy typically involves autologous stem cell transplantation (autoSCT) after induction, followed by high-dose chemotherapy treatment (2). Importantly, given the risk of the treatment and its side effect, autoSCT is usually recommended only for youngest patients, accounting for approximately 30–40% of the patients with MM. However, the majority of patients will relapse within 2–3 years from the initiation of treatment, and the overall survival (OS) is still limited (3). Nevertheless, significant advances have been made in the treatment of MM by a combination of standard chemotherapy plus novel immunomodulatory drugs (IMiDs) or proteasome inhibitors, such as lenalidomide or bortezomib (4).

Another promising approach is the immunotherapeutic treatment with natural killer (NK) cells, as they present the benefit of enhanced graft-versus-tumor (GvT) effect with a low risk of graft-versus-host disease (GVHD) [for review, see 5–7)]. Furthermore, in MM patients, an elevated number of NK cells directly correlates with a lower tumor burden (8). NK cells, which were first described by Kiessling et al., are part of the innate immune system. NK cells are highly attractive because they are not antigen specific, like T and B cells. Their activity is regulated based on the diverse expression of activating and inhibiting receptors on their surface, by which they also achieve self-tolerance (9–11). After recognition of an infected or malignant cell, NK cells can kill their target by releasing cytoplasmic perforin or granzyme, leading to death receptor-mediated apoptosis or cytokine release (12). Human NK cells are characterized by the expression of CD56 and CD16 and are divided into two distinct subtypes. The more immature subset is characterized by high expression of CD56 and low or no expression of CD16; this subset is mainly situated in the lymph nodes and secondary lymphoid tissues. After maturation, the NK cell population comprises CD56^dim^ CD16^bright^ cells and is mainly found in the bone marrow, blood and spleen (13, 14). This subset has high cytotoxic capabilities and represents approximately 90% of all NK cells in the peripheral blood (15). Furthermore, NK cells have a regulatory effect on other immune cells by secreting soluble factors, such as tumor necrosis factor alpha (TNF-α), interferon gamma (IFN-γ), granulocyte macrophage colony-stimulating factor (GM-CSF) and macrophage inflammatory proteins (MIPs) (16, 17).

For the application in immunotherapy, NK cells can be isolated from either patient or healthy donor derived PBMCs or differentiated from pluripotent stem cells. For most clinical applications, the NK cells are expanded by *ex vivo* culture. To further increase the effect of the therapy, it is important to achieve the optimal NK cell antitumor activity by using the right stimulation protocols. To date, the most common protocols stimulate NK cells with cytokines such as IL-2, IL-15 and IL-21 that induce high cytotoxicity or with IL-12, 15 and 18 to favor NK cell memory (18). Apart from stimulation with interleukins, NK cells can also be co-cultured with so-called accessory or feeder cells such as irradiated, allogeneic PBMCs or different cell lines such as K562 to further enhance NK cell expansion [for review see (18)].

A novel approach toward NK cell therapy is not only to activate them *ex vivo* but also to release the immune system from inhibition by specifically targeting immunologic checkpoints. Inhibitory receptors expressed on the NK cell surface are members of the KIR family and NKG2A. KIR receptors interact with MHC I molecules, and studies have shown that a transfer of KIR-ligand mismatched NK cells led to a lower relapse rate and a greater GvT effect due to their enhanced alloreactivity (19, 20). Moreover, several antibodies that specifically target KIR receptors have been tested or are currently in clinical trials to evaluate their efficacy against different malignancies (21).

However, due to different KIR receptor expression profiles in patients, a therapeutic targeting of selected KIR receptors could lead to a better response in some patients and a worse response in others. Moreover, the results of a clinical phase II trial testing a KIR2D specific antibody showed that treatment with the antibody led to a significant decrease in NK cell activity, directly correlating with loss of KIR2D surface expression (22). In this aspect, NKG2A could be a better therapeutic target, as it is broadly expressed on NK cells and binds specifically to HLA-E that is expressed on most malignant target cells (23). Additionally, overexpression of HLA-E in different tumors has been reported to correlate with shorter disease-free or overall survival (24, 25). In MM, HLA-E is highly expressed by primary cells, and it abolishes the overall response of NKG2A^+^ NK cells (26). Furthermore, Sarkar and colleagues postulated that the most potent NK cell subset for clinical application would be NKG2A-negative and KIR-ligand mismatched. Interestingly, NKG2A is the first inhibitory receptor that is reconstituted after SCT (27, 28). This observation might also highlight the possible relevance of NKG2A as a therapeutic target in the context of allogeneic SCT.

Overall, these findings led us to further investigate the effects of cytokine-induced NK cell activation in combination with the specific checkpoint inhibition of the NKG2A-mediated pathway as a potential strategy to optimize NK cell therapeutic approaches against MM.

## Results

### Cytokine stimulation significantly increases the NK killing ability of both patient and healthy donor NK cells against MM cell lines

**Figure 1:**
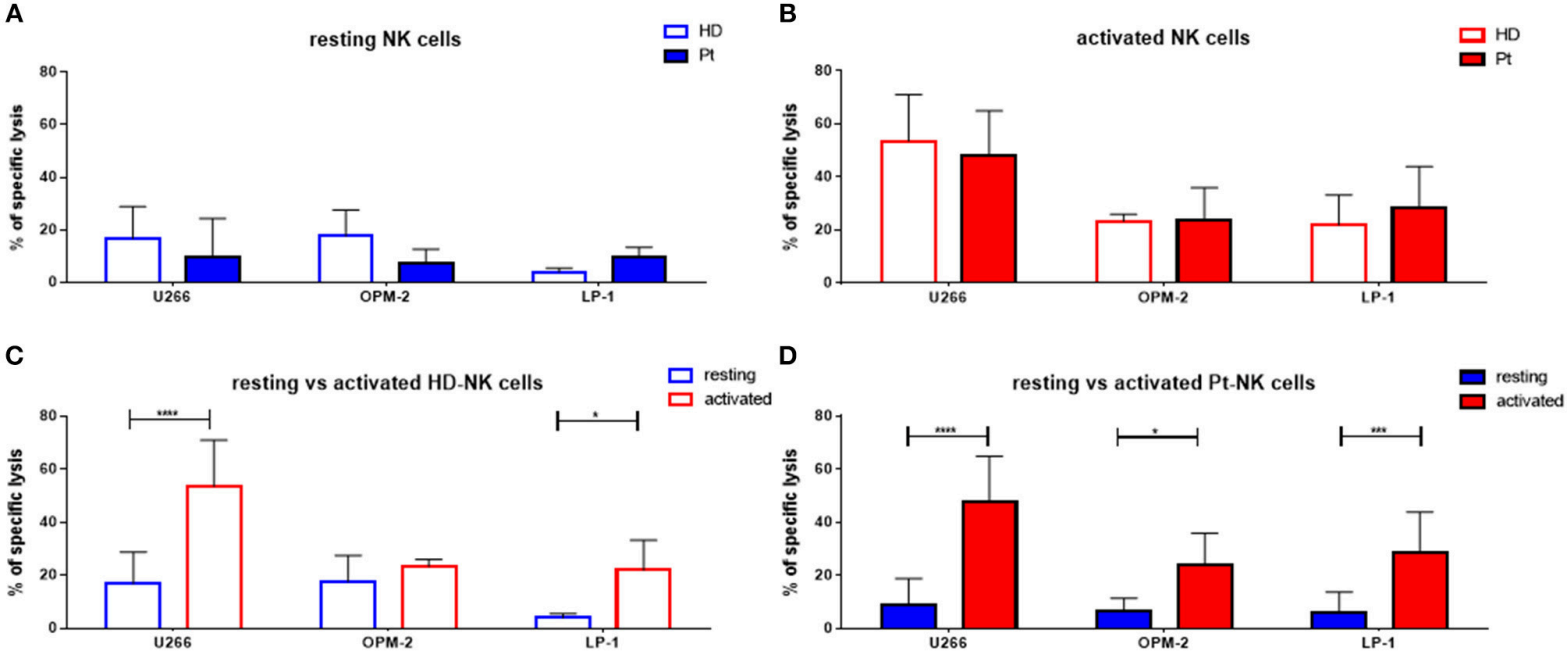
Cytokine stimulation significantly increases the NK killing ability of both patient and healthy donor NK cells against MM cell lines. **(A)** Resting and **(B)** activated (IL-2 + IL-15) NK cells isolated from patients or healthy donors were co-cultured for 24 h with the CFSE-stained multiple myeloma cell lines, LP-1, OPM-2 and U266. The percentage of dead tumor cells was determined with a live-dead stain (DAPI) via flow cytometry after 24 h of co-culture. To further demonstrate the differences in the specific lysis of tumor cells between resting and activated tumor cells, the values for NK cells isolated from **(C)** healthy donors and **(D)** patients were compared. Data on resting NK cells are depicted in blue, whereas data on activated NK cells are shown in red. Data from patient samples are shown with filled-in bars, whereas data from healthy donor samples are shown with empty bars. NK cells and tumor cells were co-cultured with a 2:1 E-T ratio. For healthy donor data, 5–6 individual experiments were performed. For patient samples, 9 individual experiments with resting NK cells and 13 individual experiments with 7 days cytokine activated NK cells were performed. Statistical analysis with two-way ANOVA + Sidak's multiple comparison: ^*^p < 0.05, ^**^p < 0.01, ^***^p < 0.001, ^****^p < 0.0001.

First, we aimed to test the “natural” ability of NK cells to kill different MM cell lines. Therefore, we isolated peripheral blood (PB) NK cells from healthy donors (HD) or untreated MM patients (Pt) at first diagnosis and co-cultured them with three different MM cell lines (U266, OPM-2, and LP-1) for 24 h (Figure 1A). The specific lysis of patient NK cells in resting conditions was approximately 10% against all three cell lines, with a trend toward reduced cytotoxic capacity compared to HD NK cells. To improve NK cell killing capacity against MM cells, we then stimulated both patient and donor NK cells for 7 days with IL-2/15 cytokine cocktail (IL-2: 100 U/ml; IL-15: 10 ng/ml) prior to performing the killing assay (Figure 1B). Of note, both patient and donor cytokine-activated NK cells showed a significantly enhanced killing capacity against the different MM cell lines compared to that of the resting NK cells (Figures 1C,D).

### Cytokine stimulation significantly increases the expression of numerous NK cell activation markers

**Figure 2:**
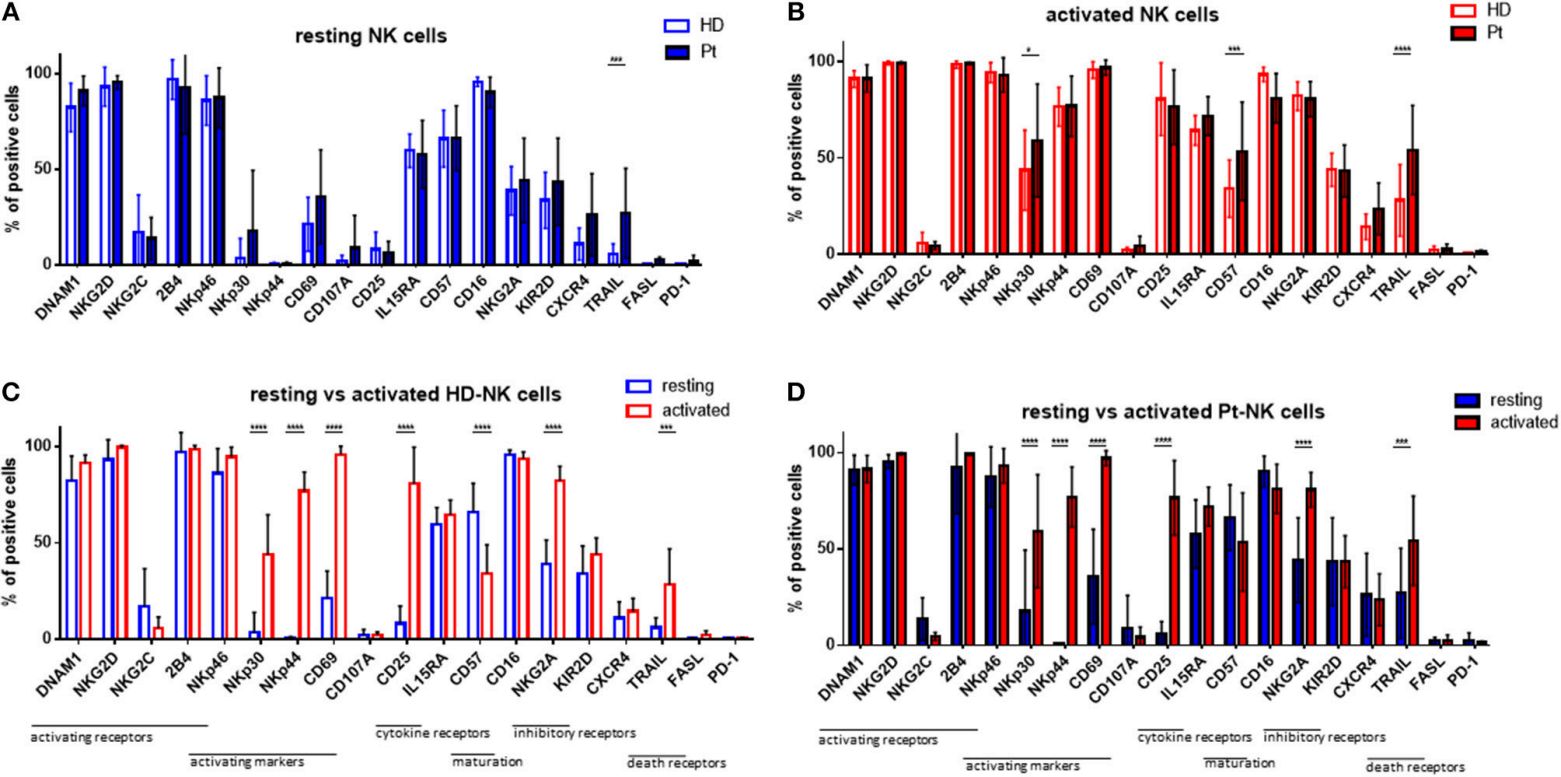
Cytokine stimulation significantly increases the expression of many NK cell activation markers. Isolated NK cells from patients and healthy donors were stained with fluorochrome-conjugated antibodies to determine surface marker expression **(A)** before and **(B)** after activation with IL-2 and Il-15. NK cell phenotypes before and after stimulation were also compared for NK cells isolated from **(C)** healthy donors and **(D)** patients. For healthy donor samples, 17 individual experiments with resting NK cells and 14 individual experiments with activated NK cells were performed (*n* = 14–17). For patient samples, 14 individual experiments with resting NK cells and 15 individual experiments with 7 days cytokine activated NK cells were performed (*n* = 14–15) Statistical analysis with two-way ANOVA + Sidak's multiple comparison: ^*^p < 0.05, ^**^p < 0.01, ^***^p < 0.001, ^****^p < 0.0001.

Given the strong and positive impact of cytokine stimulation on NK cells, we asked whether the expression of NK cell receptors and surface molecules might be modified. Therefore, we analyzed the expression levels of 19 markers by flow cytometry, including activating and inhibitory receptors, markers of activation and maturation, death receptors, homing receptors and exhaustion markers. First, we compared their expression in resting conditions in patients and healthy donors (Figure 2A). The only significant difference was the higher expression of TRAIL on patient NK cells. Next, we addressed the expression following *in vitro* cytokine stimulation for 7 days (Figure 2B). Interestingly, patient NK cells show a higher expression of activating NKp30, as well as, CD57 and TRAIL receptor. In Figure 2C the expression pattern of the 19 surface molecules before and after cytokine activation in healthy donor NK cells is depicted. Interestingly, many activating receptors and markers are strongly and significantly increased after cytokine stimulation, e.g., NKp30, NKp44, CD69, CD25, CD57, and TRAIL. This finding can explain the positive impact on the NK cell mediated killing ability of MM cells. Of note, the inhibitory receptor NKG2A is also highly upregulated after cytokine stimulation. Similar modifications have been observed for NK cells isolated from patients (Figure 2D).

### BM-derived NK cells show a similar phenotype and killing behavior to those of PB-derived NK cells in MM patients

**Figure 3:**
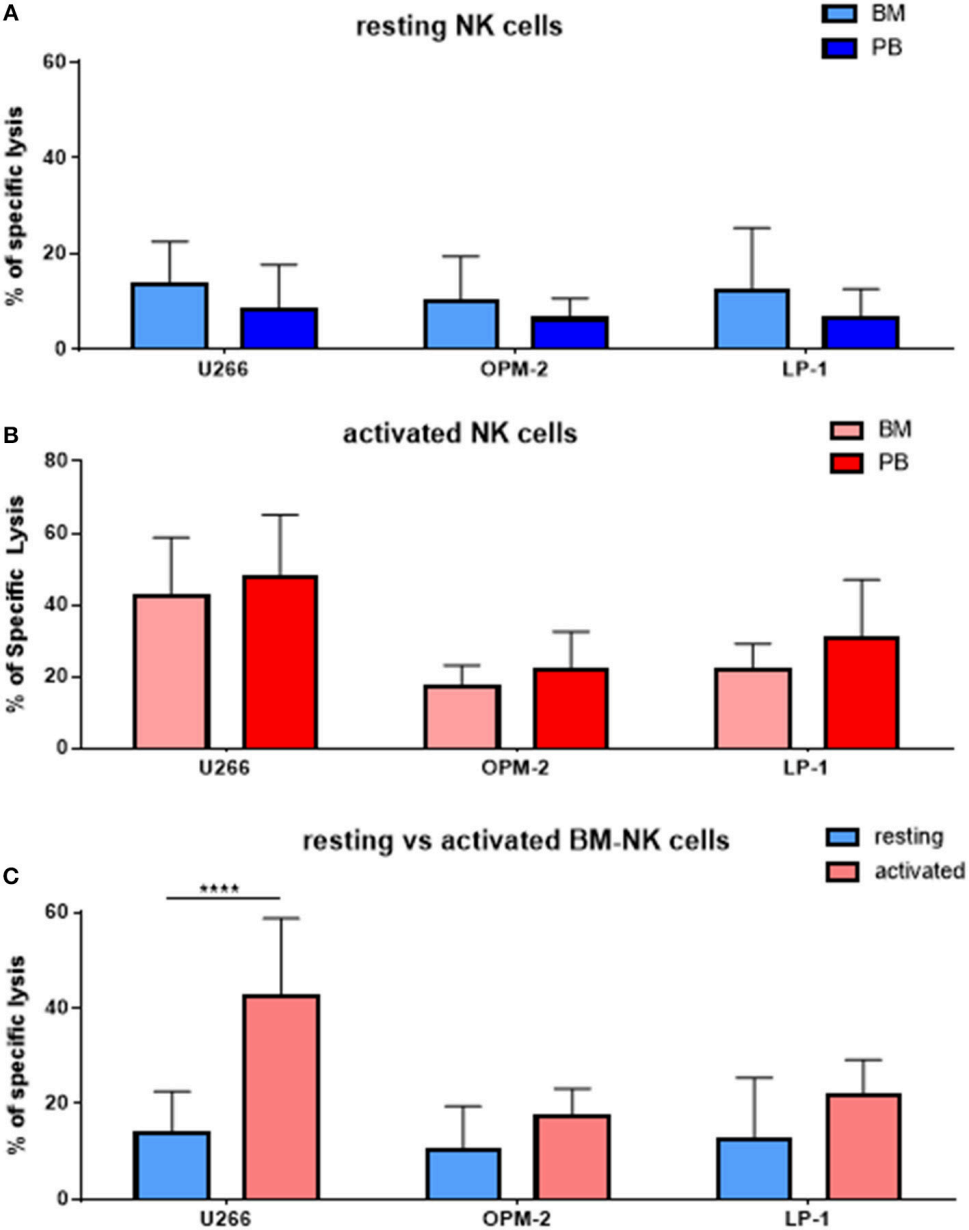
NK cells isolated from peripheral blood or bone marrow exhibit a similar cytotoxic potential and phenotype. **(A)** Resting and **(B)** activated NK cells isolated from peripheral blood and bone marrow of patients were co-cultured for 24 h with the CFSE-stained multiple myeloma cell lines LP-1, OPM-2 and U266 to compare the specific lysis of target cells. **(C)** Comparison of BM NK cytotoxic potential before and after activation. The percentage of dead tumor cells was determined with a live-dead stain (DAPI) via flow cytometry after 24 h of co-culture. For bone marrow samples, 9 individual experiments with resting NK cells and 7 individual experiments with 7 days cytokine activated NK cells were performed (*n* = 7–9). For peripheral blood samples 10 individual experiments were performed (*n* = 10). Statistical analysis with two-way ANOVA + Tukey's multiple comparison: ^*^p < 0.05, ^**^p < 0.01, ^***^p < 0.001, ^****^p < 0.0001.

**Figure 4:**
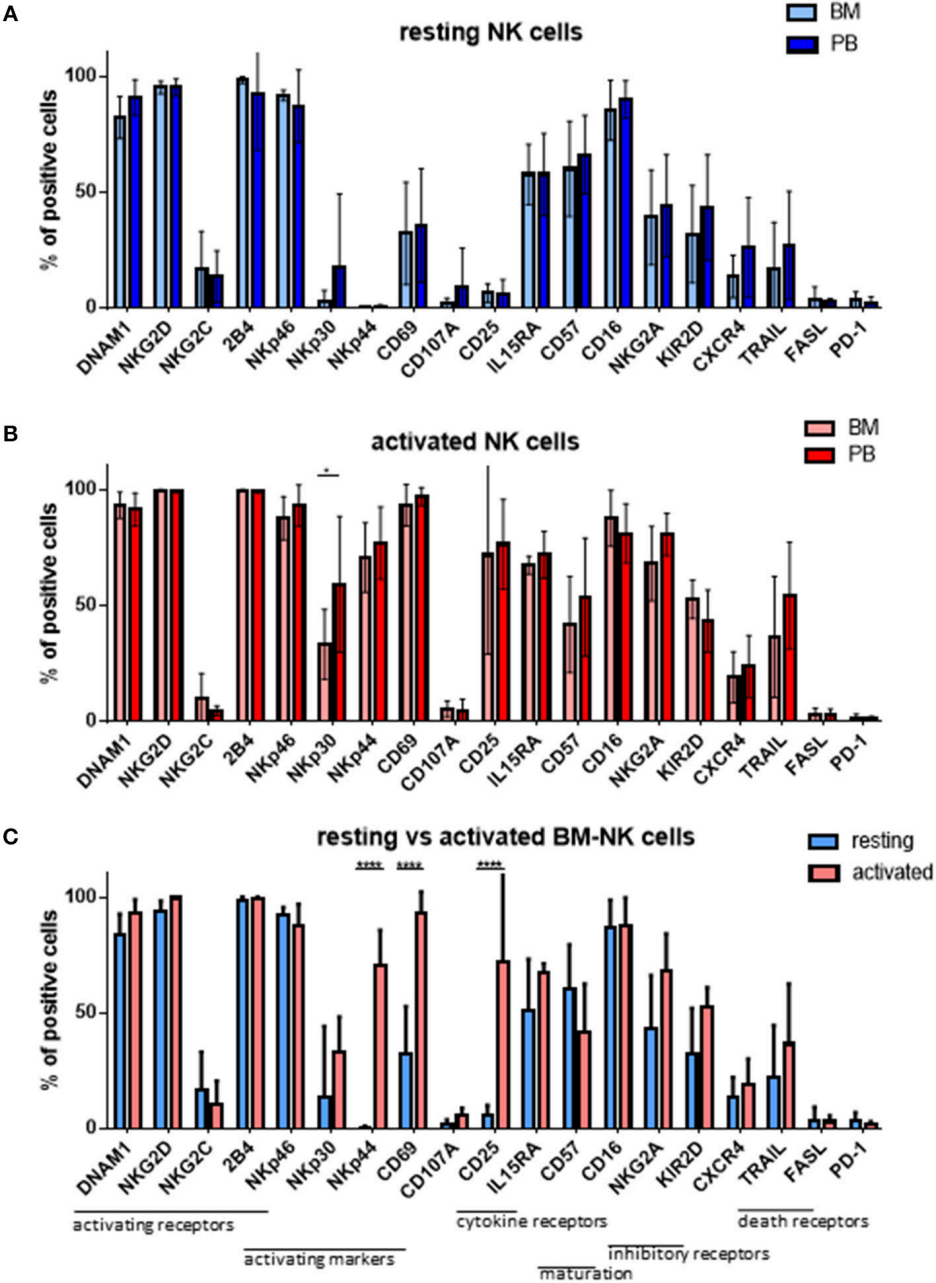
NK cells isolated from peripheral blood or bone marrow exhibit a similar cytotoxic potential and phenotype. The phenotypes of **(A)** resting and **(B)** activated NK cells isolated from peripheral blood and bone marrow of patients at TP0 were compared by staining with fluorochrome-conjugated antibodies. **(C)** Comparison of the BM NK phenotype before and after activation. For bone marrow samples, 7 individual experiments with resting NK cells and 4 individual experiments with 7 days cytokine activated NK cells were performed (*n* = 4–7). For peripheral blood samples, 14 individual experiments with resting NK cells and 15 individual experiments with activated NK cells were performed (*n* = 14–15). Statistical analysis with two-way ANOVA + Sidak's multiple comparison: ^*^p < 0.05, ^**^p < 0.01, ^***^p < 0.001, ^****^p < 0.0001.

As MM cells reside in the BM, we further asked whether and how patient NK cells derived from the bone marrow would differ from PB NK cells in newly diagnosed and still untreated MM patients (TP0). For that purpose, we performed cytotoxicity assays in resting and activating conditions with NK cell isolated from either BM or PB, as shown in Figure 3. These analyses show no significant differences in the killing activity of patient BM and PB NK cells. There is, however, a less significant increase in killing after the activation of BM NK cells, compared with PB NK cells (Figure 3C). Similarly, we also investigated the phenotype of BM NK cells. While BM NK cell receptor expression was similar to that of PB NK cells under resting conditions (Figure 4A), following cytokine stimulation, the BM NK cells showed significantly lower levels of NKp30 (Figure 4B). A comparison of the BM NK cell phenotype before and after activation showed, also in this compartment, a significant increase in the activating markers NKp44, CD69, and CD25 (Figure 4C).

### Cytokine stimulation significantly increases the NK cell killing ability of patient NK cells at various treatment points

**Figure 5:**
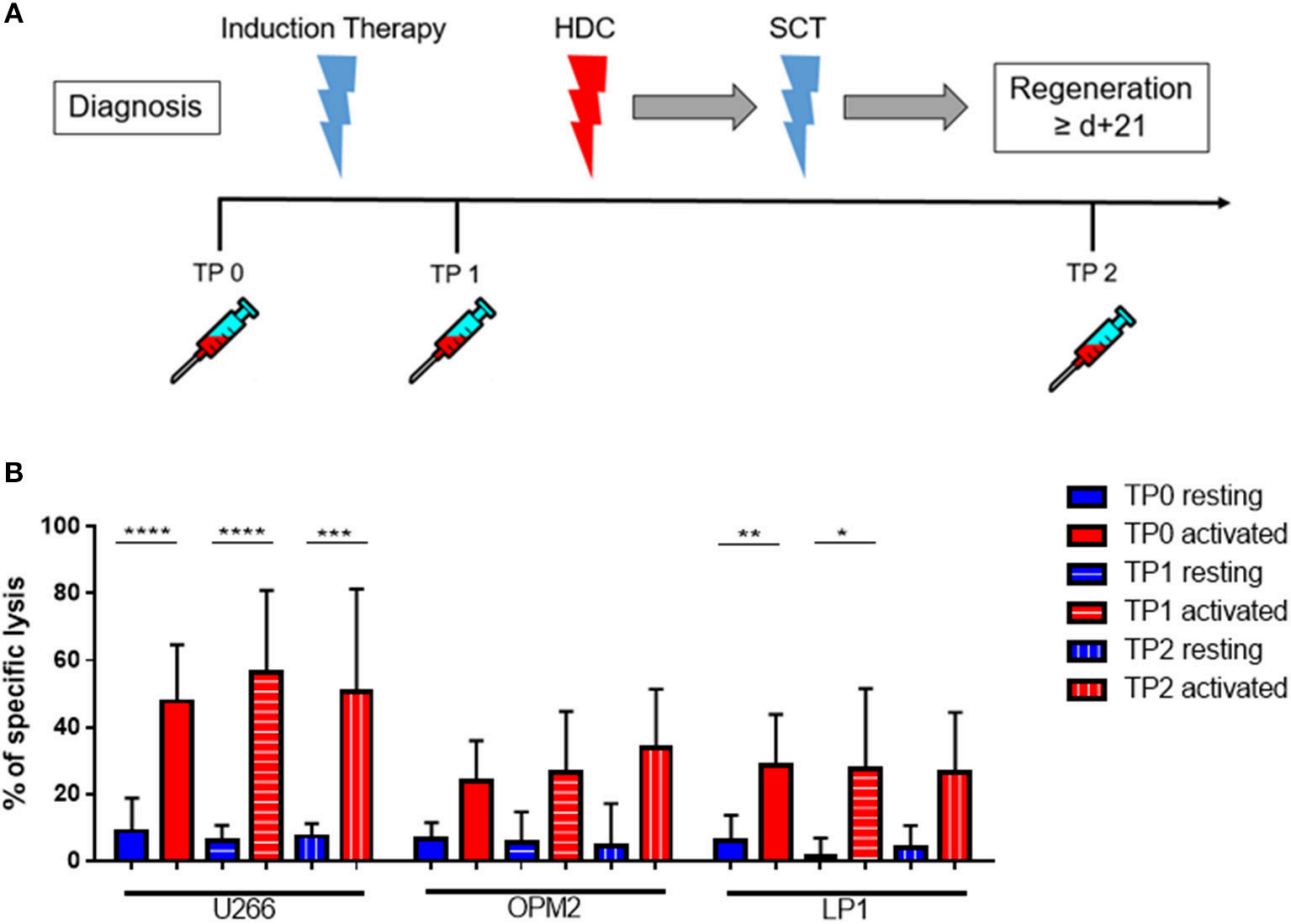
Cytokine stimulation significantly increases the NK killing ability of patient NK cells at various treatment points. **(A)** NK cells from patients were isolated at different treatment points: TP0 after diagnosis, TP1 during high-dose chemotherapy and TP2 more than 21 days after stem cell transplantation. **(B)** The isolated NK cells were co-cultured with the CFSE-stained multiple myeloma cell lines LP-1, OPM-2 and U266 either directly after isolation or after seven days of pre-activation with IL-2 and IL-15. The percentage of dead tumor cells was determined with a live-dead stain (DAPI) via flow cytometry after 24 h of co-culture. NK cells and tumor cells were co-cultured with a 2:1 E-T ratio. For TP0, 7 individual experiments with resting NK cells and 15 individual experiments with 7 days activated NK cells were performed (*n* = 7–15). For TP1, 7 individual experiments with resting NK cells and 5 individual experiments with activated NK cells were performed (*n* = 5–7). For TP2, 3 individual experiments with resting NK cells and 4 individual experiments with activated NK cells were performed (*n* = 3–4). Statistical analysis with two-way ANOVA + Tukey's multiple comparison: ^*^p < 0.05, ^**^p < 0.01, ^***^p < 0.001, ^****^p < 0.0001.

Next, we investigated whether there were differences in the killing ability of MM cells and in the cytokine susceptibility of patient NK cells at different time points (TPs) during the treatment course. Therefore, PB samples from several newly diagnosed MM patients at different treatment stages were collected, at diagnosis (TP0), after induction therapy but before high-dose chemotherapy and autoSCT (TP1) and after hematological reconstitution after autoSCT (TP2) (Figure 5A). Patient numbers and characteristics are summarized in Table 1.

**Table 1:** Patients characteristics.

**Pt. Nr**	**MM type**	**%PC–BM**	**ISS stage**	**Risk**	**Response t1**	**Response t2**
#1	lgA kappa	30	I	SR	PR	VGPR
#2	kappa	20	III	n.a.	n.a.	n.a.
#3	lgG kappa	80	III	SR	MR	VGPR
#4	lgA lambda	25	III	HR	VGPR	PR
#5	lgG kappa	40	I	SR	n.a.	n.a.
#6	lgA kappa	20	I	SR	PR	VGPR
#7	lgG kappa	20	I	SR	VGPR	CR
#8	lgG kappa	90	II	SR	PR	VGPR
#9	lgG kappa	25	I	SR	VGPR	VGPR
#10	lgG kappa	20	II	SR	VGPR	CR
#11	lgG lambda	15	I	SR	VGPR	CR
#12	lgG lambda	50	I	SR	VGPR	CR
#13	lgG kappa	30	I	HR	VGPR	n.a.
#14	lgG kappa	n.a.	III	n.a.	n.a.	n.a.
#15	lgG kappa	40	III	n.a.	n.a.	n.a.
#16	kappa	n.a.	I	n.a.	n.a.	n.a.

*Sixteen patients with newly diagnosed multiple myeloma have been included in this study. Fifteen patients had symptomatic myeloma and needed an immediate treatment. Of these 15 patients, 11 patients were treated with a bortezomib-based triplet, followed by stem cell mobilization and high-dose chemotherapy with 200 mg/m2 melphalan. The remaining 4 patients were treated with conventional dose bortezomib/ dexamethasone or lenalidomide/ dexamethasone. Responses (according to IMWG) were evaluated after induction treatment (t1), and after minimum 60 days at hematological reconstitution after high-dose chemotherapy (t2). Risk stratification delineates the following cytogenetic risk factors by FISH: high risk (HR) with presence of t_(4;14)_, or t_(14;16)_, or del(17p); standard risk (SR) with Absence of above named. PC, plasma cell; BM, bone marrow; MR, minimal response; CR, complete remission; PR, partial remission; VGPR, very good partial remission; n.a., not available*.

Cytokine-activation of NK cells significantly improved patient NK cell killing activity, especially toward the cell line U266 and LP1 (Figure 5B). There were however no significant differences in the lysis at different TPs.

**Figure 6:**
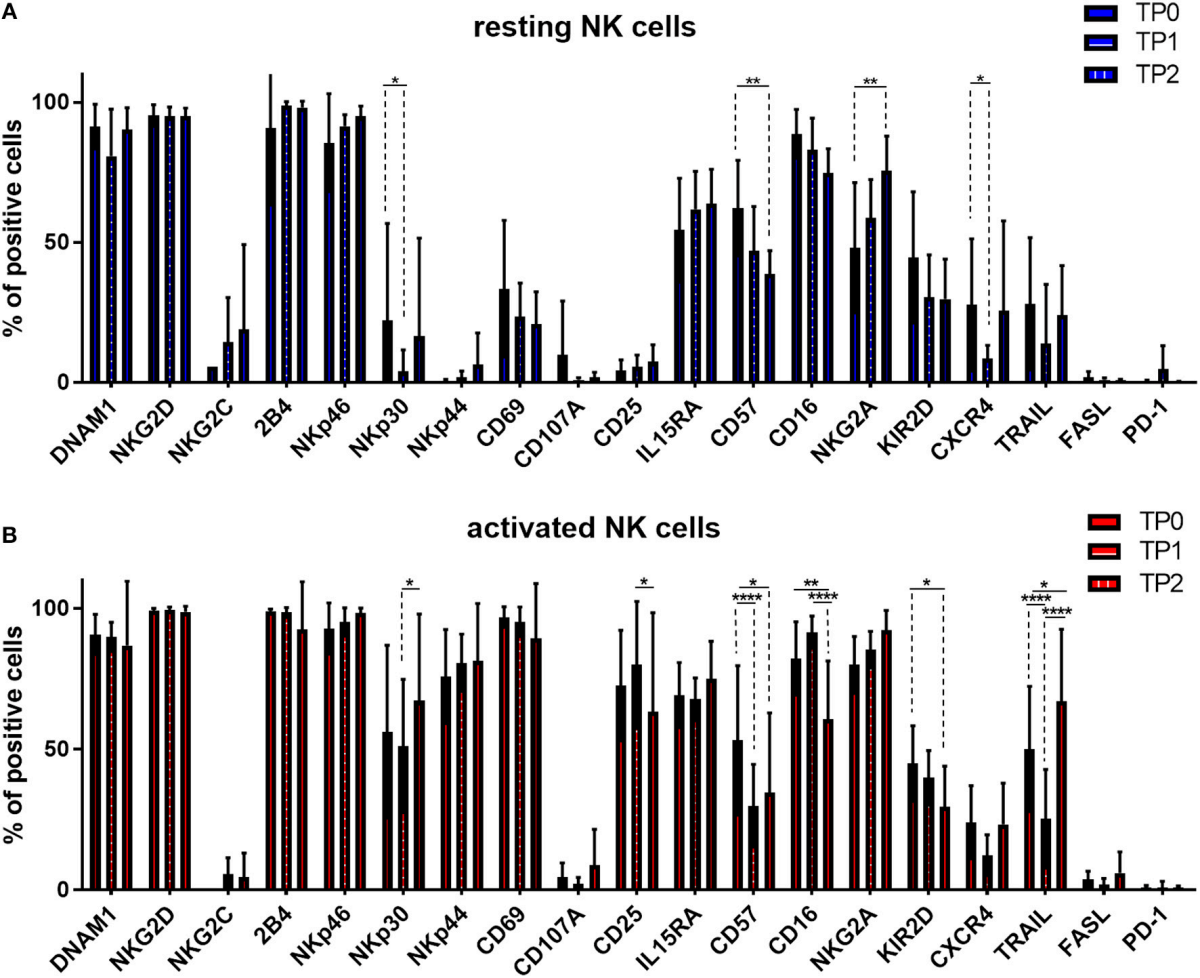
Expression of several NK cell markers significantly changed between different treatment points. NK cells from patients were isolated at different treatment points: TP0 after diagnosis, TP1 during high-dose chemotherapy and TP2 more than 21 days after stem cell transplantation. After isolation, NK cells were stained with fluorochrome-conjugated antibodies to determine surface marker expression **(A)** before and **(B)** after activation with IL-2 and Il-15. For TP0, 11 individual experiments with resting NK cells and 15 individual experiments with activated NK cells were performed (*n* = 11–15). For TP1, 10 individual experiments with resting NK cells and 16 individual experiments with 7 days activated NK cells were performed (*n* = 10–16). For TP2, 5 individual experiments with resting NK cells and 7 individual experiments with activated NK cells were performed (*n* = 5–7). Statistical analysis with two-way ANOVA + Sidak's multiple comparison: ^*^p < 0.05, ^**^p < 0.01, ^***^p < 0.001, ^****^p < 0.0001.

Moreover, we monitored the expression pattern of 19 surface molecules of interest during the course of therapy and before and after cytokine stimulation, as shown in Figure 6. The induction therapy seemed to have a slightly negative impact on the NK cell activation status in resting conditions; the activation marker NKp30 and the BM homing receptor CXCR4 are significantly downregulated at TP1 (Figure 6A). The decrease of the activating receptors NKp30 and TRAIL and the increase of NKG2A could partially explain the reduced effect of the cytokine activation on the specific lysis levels (Figure 5B). Interestingly, at the end of the therapy (TP2) and more markedly after activation, the expression levels of several markers were restored, as in the case of DNAM1, NKp30, TRAIL, and CXCR4 (Figure 6A). However, NK cells at TP2 seem to have an overall more immature phenotype due to the downregulation of CD57, CD16, and KIR2D expression and the upregulation of NKG2A expression (Figure 6). These data confirmed the downregulation of CD57, CD16, and KIR2D after therapy and in particular at TP2, as well as, the upregulation of NKG2A, indicating a more immature NK cell phenotype and possibly lower ADCC capacity of NK cells at TP2 (Figure 6B).

### Cytokine stimulation significantly increases the killing ability of MM patient NK cells even against autologous MM cells

**Figure 7:**
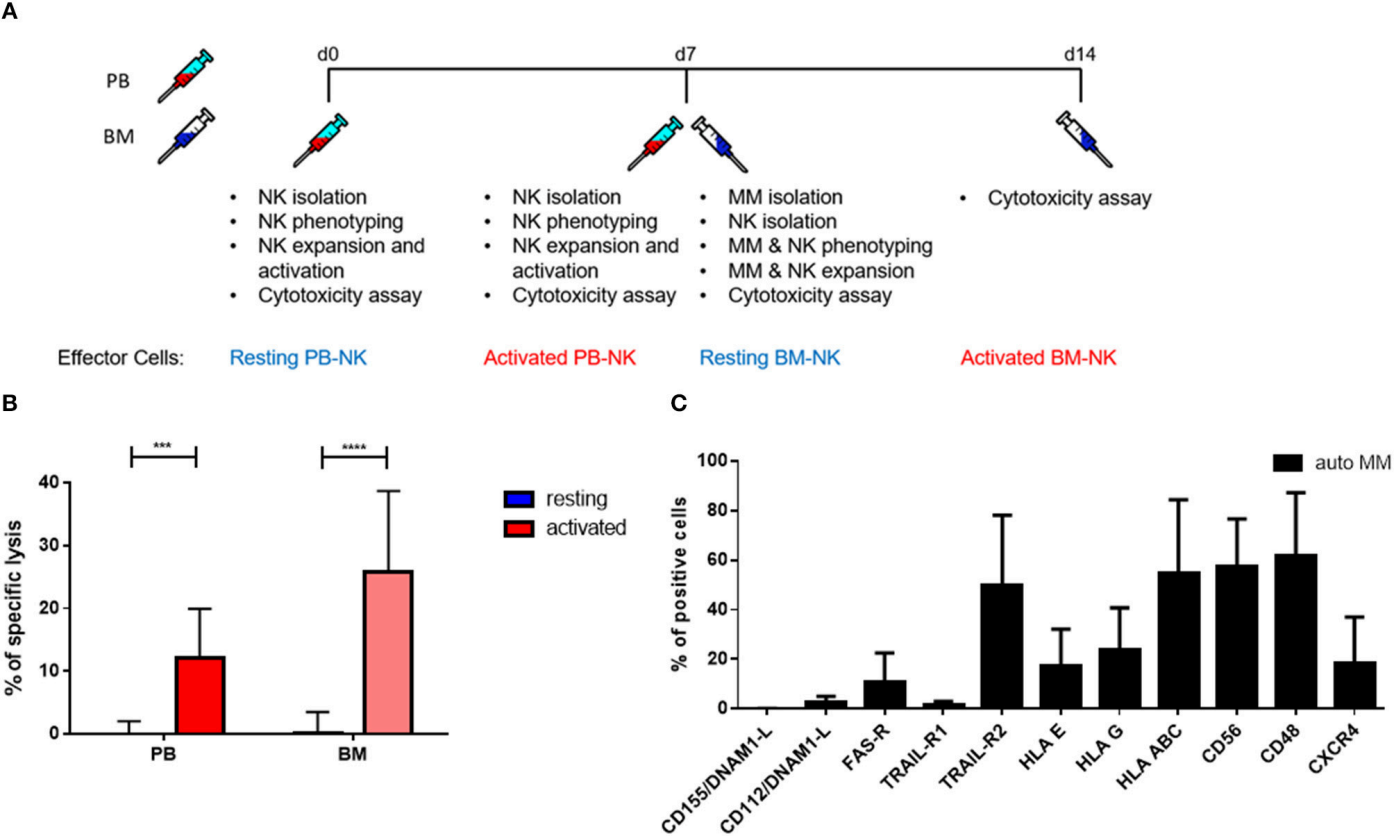
Pre-activation of NK cells significantly increases autologous tumor cell lysis. **(A)** Schematic of the experimental procedure. On d0, NK cells from peripheral blood were isolated and used for cytotoxicity assays at d0, before activation, and d7, after activation. On d7, MM cells and NK cells were isolated from BM aspirates. Those cells were also used for cytotoxicity assays at d7, before activation, and d14, after activation. **(B)** Resting and 7 days cytokine activated NK cells isolated from peripheral blood and bone marrow of patients were co-cultured for 24 h with CFSE-stained autologous multiple myeloma cells to compare the specific lysis of target cells. The percentage of dead tumor cells was determined with a live-dead stain (DAPI) via flow cytometry after 24 h of co-culture. NK cells and tumor cells were co-cultured with a 2:1 E-T ratio. **(C)** From patients, isolated primary multiple myeloma cells were stained with fluorochrome-conjugated antibodies to determine marker expression. For the bone marrow samples, 9 individual experiments with resting NK cells and 5 individual experiments with activated NK cells were performed (*n* = 5–9). For the peripheral blood samples, 9 individual experiments were performed (*n* = 9). For the phenotyping of autologous MM cells, 5 individual experiments were performed. Statistical analysis with two-way ANOVA + Tukey's multiple comparison: ^*^p < 0.05, ^**^p < 0.01, ^***^p < 0.001, ^****^p < 0.0001.

To further explore the cytotoxic potential of NK cells, we decided to study patients' killing ability against autologous tumor cells. With that aim, we isolated autologous primary tumor cells from some patients' BM aspirates that were subsequently used as a target for PB and BM NK cells from the same MM patients before and after cytokine stimulation (Figure 7A). Remarkably, both PB and BM patient NK cells in resting conditions were unable to kill autologous MM tumor cells. However, after cytokine stimulation, patient NK cells strongly and significantly increased their cytotoxic activity even against autologous MM tumor cells (Figure 7B). Of note, there were no significant differences in the killing capacity of PB and BM NK cells.

### Expression levels of NK cell receptor ligands do not correlate with differences in the susceptibility of NK cell killing

**Figure 8:**
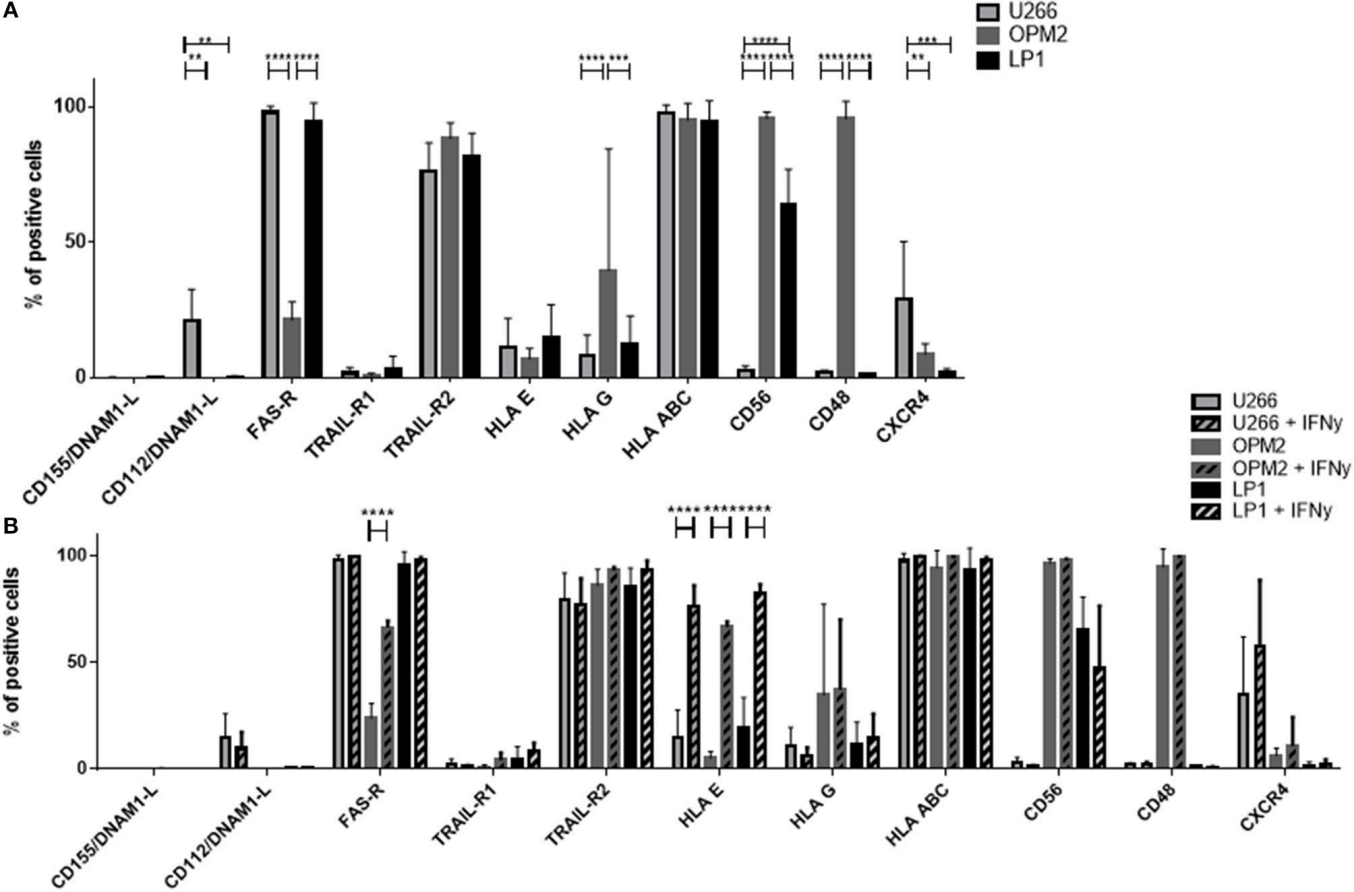
Determination of the expression of surface molecules on multiple myeloma cells. **(A)** The marker expression of different multiple myeloma cell lines was measured via FACS analysis by staining the cell lines LP-1 (black), OPM-2 (dark gray) and U266 (light gray) with fluorochrome-conjugated antibodies. **(B)** To further increase the expression of HLA-E, the multiple myeloma cell lines were incubated for 24 h with 25 nM IFN-γ before measuring surface marker expression via flow cytometry. For phenotyping the MM cells, five individual experiments were performed (*n* = 5). For phenotyping experiments after stimulation with IFN-γ, 3 individual experiments were performed (*n* = 3). Statistical analysis with two-way ANOVA + Sidak's/Tukey's multiple comparison: ^*^p < 0.05, ^**^p < 0.01, ^***^p < 0.001, ^****^p < 0.0001.

We performed a detailed flow cytometry phenotyping of the various MM target cell lines and MM primary cells to define their expression levels of 12 surface markers, with a special focus on the ligands for NK cell receptors (Figures 7C, 8). The three MM cell lines (Figure 8A) revealed differences in the expression levels of several surface markers such as FAS-R, CD56, and CD48. However, the three cell lines only slightly differed in the levels of ligands for the activating and inhibitory receptors. Notably, the phenotype did not correlate with the different levels of susceptibility to NK cell killing. Moreover, we treated the MM cell lines with IFN-γ (Figure 8B) to investigate their phenotype under pro-inflammatory conditions, and we observed one major change, namely, the strong increase in HLA-E expression.

### The combination of NKG2A blocking with cytokine stimulation further improves NK cell killing activity against MM cells

**Figure 9:**
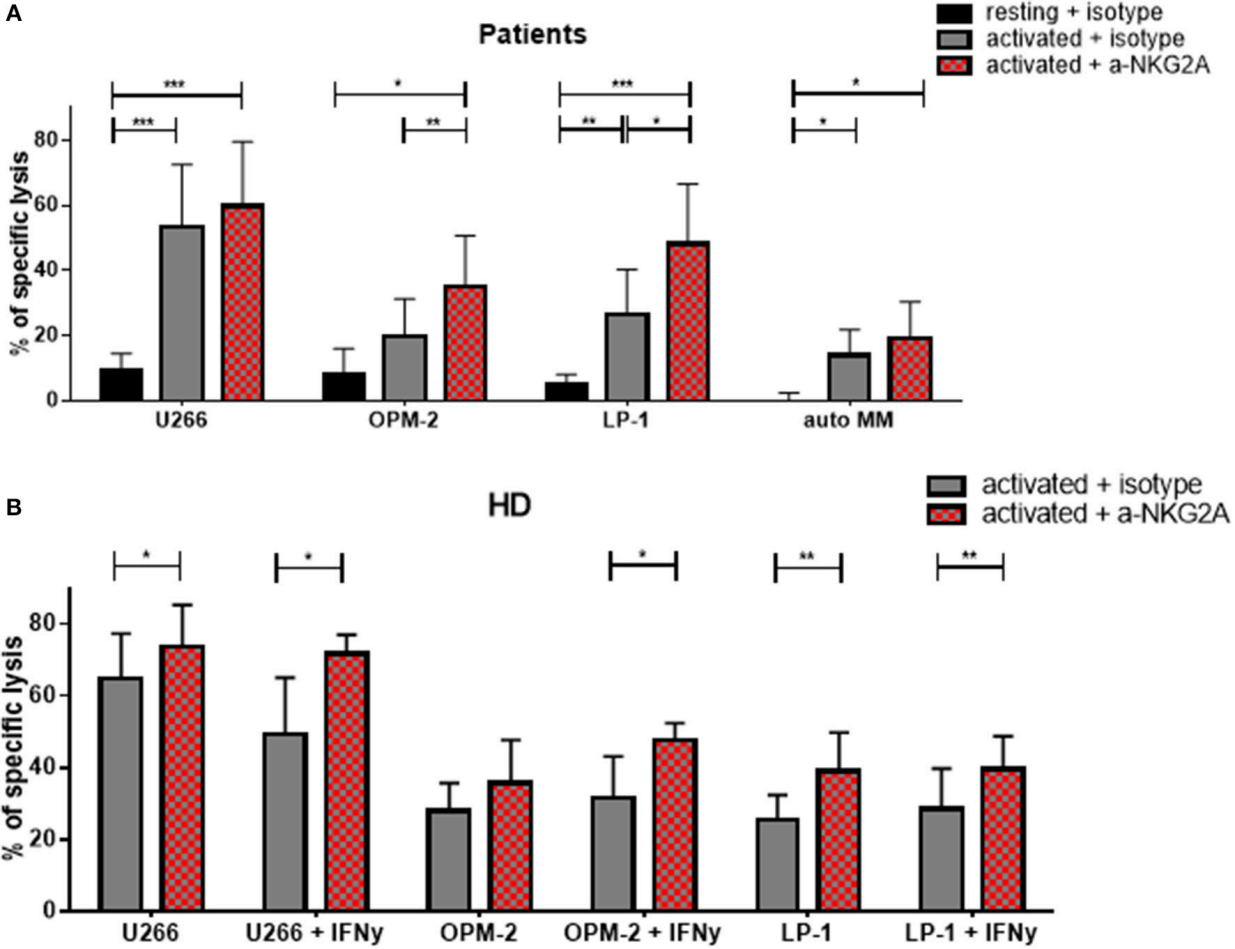
Functional blockade of NKG2A significantly increases NK cell cytotoxic potential. Isolated NK cells from **(A)** patients and **(B)** healthy donors were pre-activated by stimulating them with IL-2 and IL-15 for seven days and then co-cultured with different multiple myeloma cell lines, as well as, autologous, primary cells for 24 h. Prior to co-culture, the NK cells were treated with either an anti-NKG2A or isotype antibody to functionally block NKG2A on the NK cell surface. The percentage of dead tumor cells was determined with a live-dead stain (DAPI) via flow cytometry after 24 h of co-culture. NK cells and tumor cells were co-cultured with a 2:1 E-T ratio. For patient samples, 8 individual experiments for resting + isotype, 9 individual experiments for activated + isotype and activated + a-NKG2A were performed (*n* = 8–9). For healthy donor samples, 5 individual experiments were performed (*n* = 5). Statistical analysis with two-way ANOVA + Sidak's multiple comparison: ^*^p < 0.05, ^**^p < 0.01, ^***^p < 0.001, ^****^p < 0.0001.

Given that the inhibitory receptor NKG2A was the only inhibitory receptor upregulated following cytokine stimulation, we hypothesized that blocking this inhibitory NK cell checkpoint could further improve the lysis of MM target cells by NK cells (Figure 9). Therefore, NK cells from patients were incubated with a blocking antibody or the control isotype prior to the cytotoxicity assay (Figure 9A). Importantly, blocking NKG2A resulted in a clear increase in the NK cell-mediated killing of OPM-2 and LP-1 cell lines. Hoping to achieve the possible use of third party or donor NK cells for adoptive therapy in MM, we examined whether the same effect could be seen using HD NK cells. HD NK cell show a significant better lysis against the targets U266 and LP-1. Importantly, the improvement of NK cell mediated killing capacity was even stronger when the MM cell lines were previously treated with IFN-γ and were highly HLA-E positive (Figure 9B).

## Discussion

MM is a highly aggressive plasma cell neoplastic disorder, and despite recent therapeutic advancements, it is still considered an incurable disease. Immunotherapy, including the use of cytokines, checkpoint inhibitors or cellular immunotherapeutics, holds great new promises to expand anti-myeloma treatment options. Because of its given antitumor activity, adoptive NK cell-based immunotherapy represents a potential treatment approach for myeloma patients.

In our study, we demonstrated that NK cells activated with IL-2 and IL-15 were able to efficiently kill multiple myeloma cell lines and autologous myeloma cells. Importantly, their killing activity was independent of the NK cell source from PB or BM or the time point of their isolation during myeloma treatment. Remarkably, their anti-myeloma activity could be further enhanced by an NKG2A checkpoint blockade.

Initially, it has been reported that NK cells from myeloma patients have a defect in their cytotoxic activity (29–31) and that myeloma cells are becoming more resistant to NK cell killing during disease progression (32). As an example of tumor immune evasion strategies it has been reported that high TGF-β levels in the tumor environment decrease the ability of NK cells to respond to IL-12 and IL-15 (33). On the other side, expression of IL-15 receptor and autocrine production of IL-15 has been suggested as mechanism of tumor propagation in MM (34). Moreover, the expression of activating NK cell receptors (e.g., NCR, NKG2D, 2B4, DNAM1) is known to be decreased in MM patients (35, 36). Although our data only demonstrated a difference in membrane bound TRAIL expression on resting NK cells of healthy donors and MM patients, the NK cell mediated cytotoxic activity against myeloma cell lines was only marginal.

However, upon *ex vivo* cytokine stimulation, the cytotoxicity of NK cells against multiple myeloma cell lines significantly improved. Furthermore, autologous inhibition was overcome and led to increased anti-myeloma activity even against autologous, primary myeloma cells. This result is in line with other studies investigating *ex vivo* NK cell expansion/activation protocols for anti-myeloma immunotherapy.

One study established a GMP-compliant protocol to expand NK cells from MM patients using IL-2 and anti-CD3 for 20 days, leading to a sufficient NK cell expansion of an average 511-fold (37). Importantly, only activated and expanded NK cells were able to kill autologous myeloma cells *in vitro*, without any cytotoxicity against autologous CD34^+^ cells. This effect was time dependent since the NK cells' anti-myeloma activity was only marginal after a 5-day stimulation period.

Another approach was to use a genetically modified K562 cell line expressing the 41BB-ligand and IL-15. NK cells from healthy donors and myeloma patients were successfully expanded and able to kill allogeneic and autologous primary myeloma cells *in vitro* and in an *in vivo* mouse model (38). Again, only expanded NK cells demonstrated a significant killing activity against myeloma cells, whereas non-expanded NK cells did not. In accordance with our data, expanded NK cells from HD and MM patients demonstrated similar cytotoxic activities against allogeneic myeloma targets, indicating that *ex vivo* cytokine stimulation is able to overcome the NK cell cytotoxicity defects of myeloma patients.

The mechanism behind their improved cytotoxicity has been attributed to the increased expression of activating NK cell receptors (e.g., NKG2D, DNAM1, and NCRs) and cytotoxic effector molecules (e.g., granzyme B and perforin), as well as, membrane bound death receptor ligands (e.g., TRAIL). Their contributions have been demonstrated by performing blocking experiments upon NK cell cytokine stimulation (32, 38, 39), which is of particular importance since anti-myeloma drugs are known to increase activating or decrease inhibitory NK cell receptor ligands on myeloma cells (40–42). We observed an increase in the surface expression of activating receptors upon cytokine stimulation. In addition, TRAIL expression was significantly increased upon cytokine stimulation on myeloma patients' NK cells, which is in concordance with the results of previous reports demonstrating TRAIL upregulation and improved NK cell cytotoxicity upon IL-2 and/or IL-15 stimulation (43, 44). Based on this knowledge, the first clinical trials using adoptive NK cell transfer to treat myeloma patients have been completed. While one trial used the allogeneic NK cell line, NK-92, within a phase-I dose-escalating trial for treating refractory hematological malignancies in a non-transplantation setting (45), two other trials were performed within an autoSCT setting. The first used haploidentical, KIR-ligand mismatched NK cells expanded with IL-2 and anti-CD3, demonstrating the safe engraftment of autologous stem cells with no signs of GVHD in treated myeloma patients (46). Similar safety results were obtained in a second trial expanding NK cells using irradiated K562 cells expressing membrane bound IL-21 in combination with IL-2 (47).

Based on these first in-human trials, we investigated the best time point for harvesting NK cells to expand them for adoptive NK cell transfer within an autoSCT setting. Interestingly, in this study independent of prior treatments with the proteasome-inhibitor bortezomib, immune modulating substances (IMiDs) such as lenalidomide or chemotherapeutics including melphalan, NK cells isolated at all chosen TPs demonstrated similar cytotoxic activity against myeloma cell lines. This result was surprising as an NK cell activating effect of IMiDs and a more inhibitory effect of proteasome inhibitors have been previously reported (for review see (48)). Furthermore, despite the differential expression of activating and inhibitory receptors in resting NK cells, especially the upregulation of NKG2A expression and the downregulation of CD57 expression at TP2, indicate a more immature NK cell phenotype, in line with earlier reports (28). In addition, lower TRAIL expression upon cytokine expression on NK cell from TP1 did not seem to have a negative influence on their anti-myeloma activity.

Therefore, we propose that NK cells can be freshly isolated before the start of high-dose chemotherapy (HD) and autoSCT in order to be activated, expanded and re-infused before or after autoSCT. Using freshly isolated NK cells is of importance since adoptive NK cell transfer studies have demonstrated that the use of fresh NK cells may be more beneficial than using cryopreserved cells (49, 50).

Although we observed increased anti-myeloma activity and expression of activating NK cell receptors, there was a significant upregulation of the inhibitory receptor NKG2A, while other receptors such as KIR2D or PD1 were not upregulated. This result is in contrast to other reports, which have demonstrated the strong upregulation of PD1 upon cytokine stimulation (51). In addition, CD57 expression was downregulated on our activated NK cells, corresponding to a more immature phenotype (52) as has been described by others. In addition, also CD16 was downregulated which might reduce the NK cell ADCC capacity. Despite their more immature phenotype, those NK cells were multifunctional since they demonstrated increased cytokine production, proliferation capacity and degranulation upon target cell recognition. In addition, cytotoxic molecules were strongly upregulated (51). These data indicate that the classical view of NKG2A^+^ NK cells as being more immature and the main cytokine producers does not hold true when the cells are stimulated with cytokines.

NKG2A is known to interact with HLA-E and is known to be increased on myeloma cell lines. Although HLA-E was low on our myeloma cell lines, its expression was increased on primary myeloma cells. This finding is in line with other reports demonstrating higher levels of HLA-E on primary myeloma cells than on cell lines, which can be upregulated upon *in vivo* transfer (26). In addition, HLA-E expression was upregulated upon IFN-γ treatment, diminishing their susceptibility toward NK cell treatment, which was reverted upon blocking the NKG2A-/HLA-E interaction. The importance of the NKG2A/HLA-E interaction has been demonstrated for other diseases, e.g., within a humanized, post-transplantation model, which demonstrated that re-constituted NKG2A^+^ NK cells were able to kill human primary leukemia cells when mice were injected with an anti-human NKG2A antibody (53). In addition, blocking the NKG2A/HLA-E interaction was able to restore NK cell dysfunction against CLL cells (54). This finding has led to different clinical phase 1 trials to evaluate the efficacy and safety of the NKG2A blockade in patients with CLL disease (NCT02557516) or in a post-allogeneic SCT setting (NCT02921685).

In summary, our data provide evidence for the use of *ex vivo* cytokine-activated NK cells as an immunotherapy to treat myeloma patients within an autoSCT setting in combination with NKG2A blockade.

## Materials and methods

### NK cell isolation and culture

Blood samples were obtained from healthy donors or from diagnosed MM patients in accordance with the Declaration of Helsinki. All subjects provided written, informed consent. This study was carried out in accordance with the recommendations of the Ethics Committee of the University Hospital Frankfurt. The protocol of this study (SHN-02-2015) was approved by the above mentioned committee. Patient numbers and characteristics are summarized in Table 1. Patient selection was randomly at the onset of MM diagnosis prior to any treatment. The age range for patients was from 45 to 70 years, but only from 29 to 64 years for healthy donors.

Blood from patients was collected at three different treatment points (TP0 after diagnosis, TP1 in the pause before high-dose chemotherapy and autoSCT, TP2 >21 d after SCT). Peripheral and bone marrow blood mononuclear cells were isolated by Ficoll density gradient centrifugation (Biochrom, #L6115). NK cells were enriched by negative selection of NK cells with an EasySep NK Cell Enrichment Kit (StemCell Technologies, #19055) according to the manufacturer's protocol. NK cell purity was assessed by flow cytometry and was >90% with the used isolation method. Cells were cultured in X-VIVO 10 (Lonza, #BE04-743Q) medium supplemented with 5% heat-inactivated human plasma (DRK Blutspendedienst) and 1% penicillin with streptomycin (Invitrogen, #15140-122). NK cells were expanded *ex vivo* by additionally supplementing the medium with IL-2 (100 U/ml) (Peprotech, #200-02) and IL-15 (10 ng/ml) (Peprotech, #200-15). Every third day 80 μl of old medium were removed and100 μl of fresh medium with cytokines were added to the wells. All cells were maintained at 37°C with 5% CO_2_ atmospheric conditions. Unless otherwise stated, NK cells were used in the experiments after 7 days of culture with IL-2/15.

### Culture and treatment of MM cells

Autologous MM cells were isolated from bone marrow aspirates using CD138 MicroBeads and a MidiMACS Separator (Miltenyi Biotech, #130-042-30, #130-097-614). Cells were cultured in X-VIVO 10 (Lonza, #BE04-743Q) medium supplemented with 5% heat-inactivated human plasma (DRK Blutspendedienst) and 1% penicillin with streptomycin. Multiple Myeloma cell line originally were received from the DSMZ (Leibniz-Institut DSMZ-German Collection of Micro-organisms and Cell Cultures GmbH). To further confirm the identity of the cell lines we performed phenotypic flow cytometry analysis (Supplemental Figures 4, 6), HLA-ABC genotyping (Supplemental Table 1) and and short tandem repeat (STR) analysis of the cell line U266 (Supplemental Table 2). Taken all the data together, we could confirm the identity of our MM cell lines by different orthogonal methods, demonstrating therefore absence of cross-contamination. The MM cell line LP-1 was cultured in IMDM supplemented with 10% heat-inactivated fetal bovine serum and 1% penicillin and streptomycin and glutamine. The cell lines OPM-2 and U266 were cultured in RPMI 1640 + Glutamax (Life Technologies, #31870-025) supplemented with 10% heat-inactivated fetal bovine serum and 1% penicillin with streptomycin. All cells were maintained at 37°C with 5% CO_2_ atmospheric conditions.

### FACS phenotyping and purity check

For the flow cytometric measurements, 1 × 10^5^-10^6^ cells were used per reaction tube. Cells were stained for 20 min at 4°C. For NK cell phenotyping experiments, cells were stained with the following antibodies: 7AAD PerCP (#559925), CD107a APC-H7 (#561343), CD184 PE-Cy7 (#560669), CD226 FITC (#559788), CD25 BV605 (#562660), CD335 PE (#331908), CD56 BV421 (#562751), CD69 BV605 (#562989) (all from BD Biosciences), CD16 APC ALEXA700 (#302025), CD19 PerCP (#302228), CD215 APC (# 330209), CD253 PE (# 308206), CD3 PerCP (# 300428), CD336 PE (# 558563), CD57 APC (# 322314) (all from Biolegend), CD138 BV510 (# 130-101-169), CD14 PerCP (# 130-094-969), CD244 PE-Cy7 (# 130-099-074), CD279 PE (# 130-096-164), KIR2D FITC (# 130-098-689) (all from Miltenyi Biotech), CD159a PE (PNIM3291U), CD314 APC (# A22329) (both from Beckman Coulter) and CD159c ALEXA488 (# FAB138G-100), CD337 ALEXA488 (# FAB1849G) (both from R&D). Purity of the isolated NK cells was checked with the following: DAPI (AppliChem), CD56 FITC (# 345811) (BD Biosciences), CD16 APC ALEXA700 (# 302025), CD19 PerCP, CD3 APC (# 300412) (all from Biolegend), CD45 PE (# MHCD4504) (Invitrogen) and CD138 BV510 (Miltenyi Biotech). The NK cell gating strategy for all 19 surface markers that have been analyzed by flow cytometry is depicted in Supplemental Figures 1, 2 performed on a representative healthy donor sample. Dead NK cells have been excluded by 7AAD staining, and gates defining the positive percentage of NK cell populations were set using positive, negative and internal controls (Supplemental Figure 5).

For MM cell phenotyping cells were stained with the following antibodies: CD184 PE-Cy7, CD56 BV421 (BD Biosciences), CD112 PE, CD261 PE, CD262 APC, CD95 BV412, HLA-ABC BV605, HLA-E PE-Cy7, HLA-G APC (Biolegend), CD138 BV510, CD48 APC-H7 (Miltenyi), and CD155 FITC (R&D). Dead cells have been excluded by DAPI-expression. The gating strategy for all 11 surface markers that have been analyzed by flow cytometry is depicted in Supplemental Figure 4. Gates defining the positive percentage of MM cells expressing a specific surface marker were set using positive, negative and internal controls (Supplemental Figure 6).

The purity of the isolated, autologous MM cells was determined with the following antibodies: CD184 PE-Cy7, (BD Biosciences), CD45 FITC (#6603838), CD56 APC (#IM2474) (both from Beckmann Coulter), CD19 PerCP (Biolegend), CD38 PE (DakoCytomation), and CD138 BV510 (Miltenyi Biotech). MM cells were defined as DAPI-, CD45-/low, CD19-, CD138^+^ and CD38^+^ as shown in Supplemental Figure 3. Of note, CD138 staining following MM cell isolation was often low due to the rapid internalization and the possible competition with the CD138-beads used for the positive selection procedure.

### Analysis of tumor cell death

MM tumor cells were stained with the Cell Trace^TM^ Cell Proliferation Kit (Invitrogen, #C34554), resuspended in X-VIVO 10 media supplemented with 5% heat-inactivated human plasma and 1% penicillin with streptomycin and seeded in a 96 V-bottom well plate. NK cells were seeded into the corresponding wells. In experiments where activated NK cells were used, the medium was additionally supplemented with IL-2 (100 U/ml) and IL-15 (10 ng/ml). All experiments were performed with an E:T ratio of 2:1. The 96-well plates were incubated for 24 h at 37°C with 5% CO_2_ atmospheric conditions. After incubation, the cells were resuspended and transferred to FACS tubes. Directly before measurement, 250 μl DAPI solution (DAPI 1:6000 in PBS) (AppliChem, #A4099,0010) was added to the tubes and incubated for 3 min. For each combination, two wells were filled, representing technical replicates. In addition, control wells for all tumor targets used in the experiment were added, containing target cells only in order to determine the spontaneous lysis. In the final evaluation of the experiment, the specific lysis was calculated as the percentage of dead tumor cells in the wells containing target and effector cells minus the spontaneous lysis of the respective tumor cell condition. Through this calculation of the specific lysis as the percentage of killed tumor cells was attributed completely to the NK cell effector function. Dead target cells were calculated as Cell Trace+ DAPI+ cells.

### Blocking experiments

For blocking experiments NK cells were incubated for at least 30 min with 30 μg/ml blocking antibody prior to co-culture. Blocking experiments were performed with an anti-NKG2A antibody (Beckman Coulter, #IM2750). As control a Purified Mouse IgG2b isotype (Biolegend, #400302) was used.

## Author contributions

ST, IvM, and EU designed the project. ST, SW, and BR performed the experiments. ST and SW analyzed the data. ST, SW, HS, PB, IvM, BJ, and EU discussed the data. ST, SW, and EU wrote the manuscript with the contribution of all other co-authors.

### Conflict of interest statement

The authors declare that the research was conducted in the absence of any commercial or financial relationships that could be construed as a potential conflict of interest.

## Abbreviations

MM: multiple myeloma
CD: cluster of differentiation
CT: chemotherapy
DAMPs: damage associated molecular patterns
DCs: dendritic cells
DNA: deoxyribonucleic acid
GM-CSF: granulocyte macrophage colony-stimulating factor
HD: healthy donors
NK cells: natural killer cells
IFN: Interferon
Il: Interleukin
ns: not significant
Pt: patients
PB: peripheral blood
BM: bone marrow
TP0: treatment point 0
TP1: treatment point 1
TP2: treatment point 2
FACS: fluorescence activated cell sorting
IL15RA: IL-15 receptor alpha.
