# Supplementary material for: Enhancing the Activation and Releasing the Brakes: A Double Hit Strategy to Improve NK Cell Cytotoxicity Against Multiple Myeloma
Source: Front Immunol. 2018 Nov 27;9:2743. doi: 10.3389/fimmu.2018.02743 (PMC6277768; doi:10.3389/fimmu.2018.02743)
Supplement: Supplementary file 1 [file Data_Sheet_1.PDF]

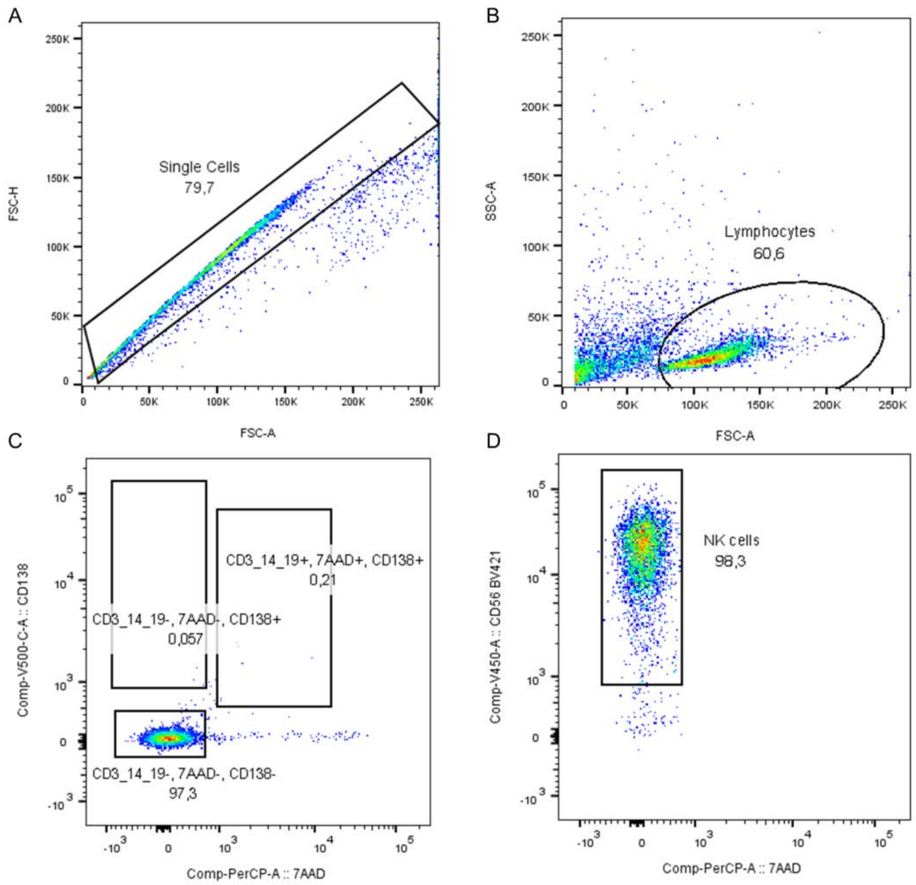

Supplemental Figure 1: Background gating strategy for NK cell experiments.

A) Only single cells were included in further analysis. B) The distinct lymphocyte population was gated. C) Exclusion of $CD3/14/19^{+}$, $7AAD^{+}$ and $CD138^{+}$ cells. D) NK cells were defined as $CD56^{+}$ and $7AAD^{-}$.

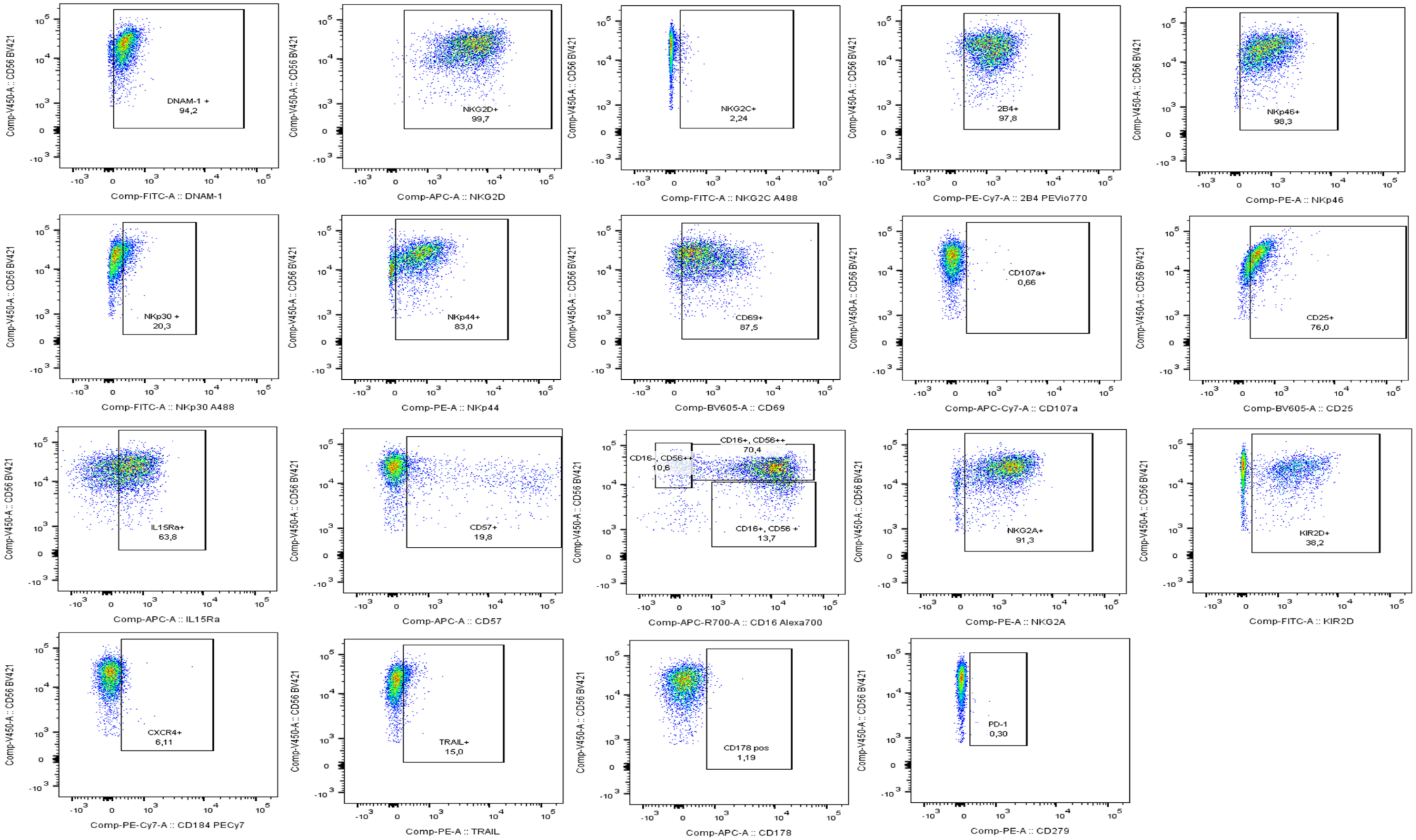

Supplemental Figure 2: NK cell phenotyping marker overview on cytokine activated PB NK cells.
Representative flow cytometry gating for all 19 cell markers that have been analyzed.

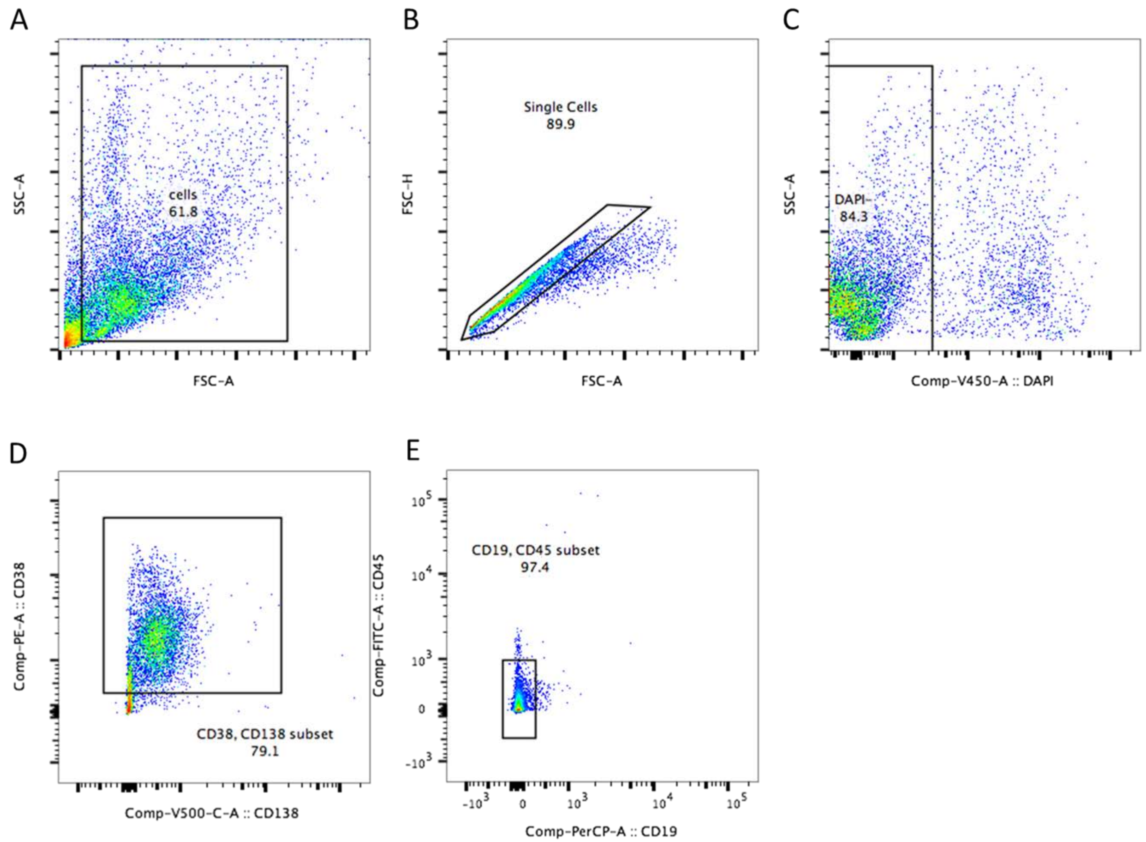

Supplemental Figure 3: Background gating strategy for multiple myeloma cell experiments. A) Exclusion of debris. B) Exclusion of doublets. C) Exclusion of DAPI⁺ cells. D) Selection of CD38 and CD138 expressing cells. E) Exclusion of CD19⁺ and CD45⁺ cells.

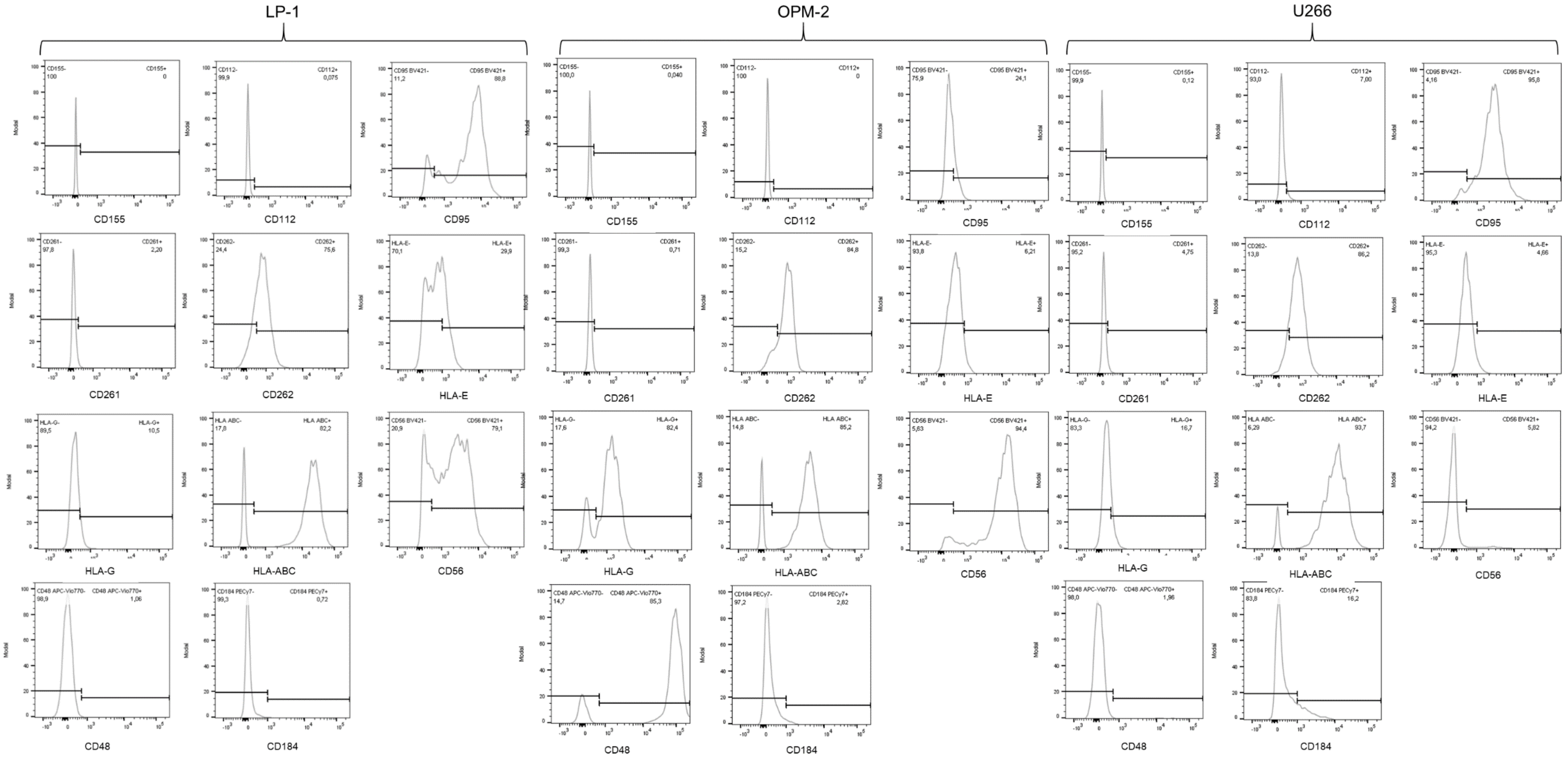

Supplemental Figure 4: Multiple myeloma phenotyping marker overview. Representative flow cytometry gating for all 11 cell markers that were used for the phenotyping analysis of the three multiple myeloma cell lines LP-1, OPM-2 and U266.

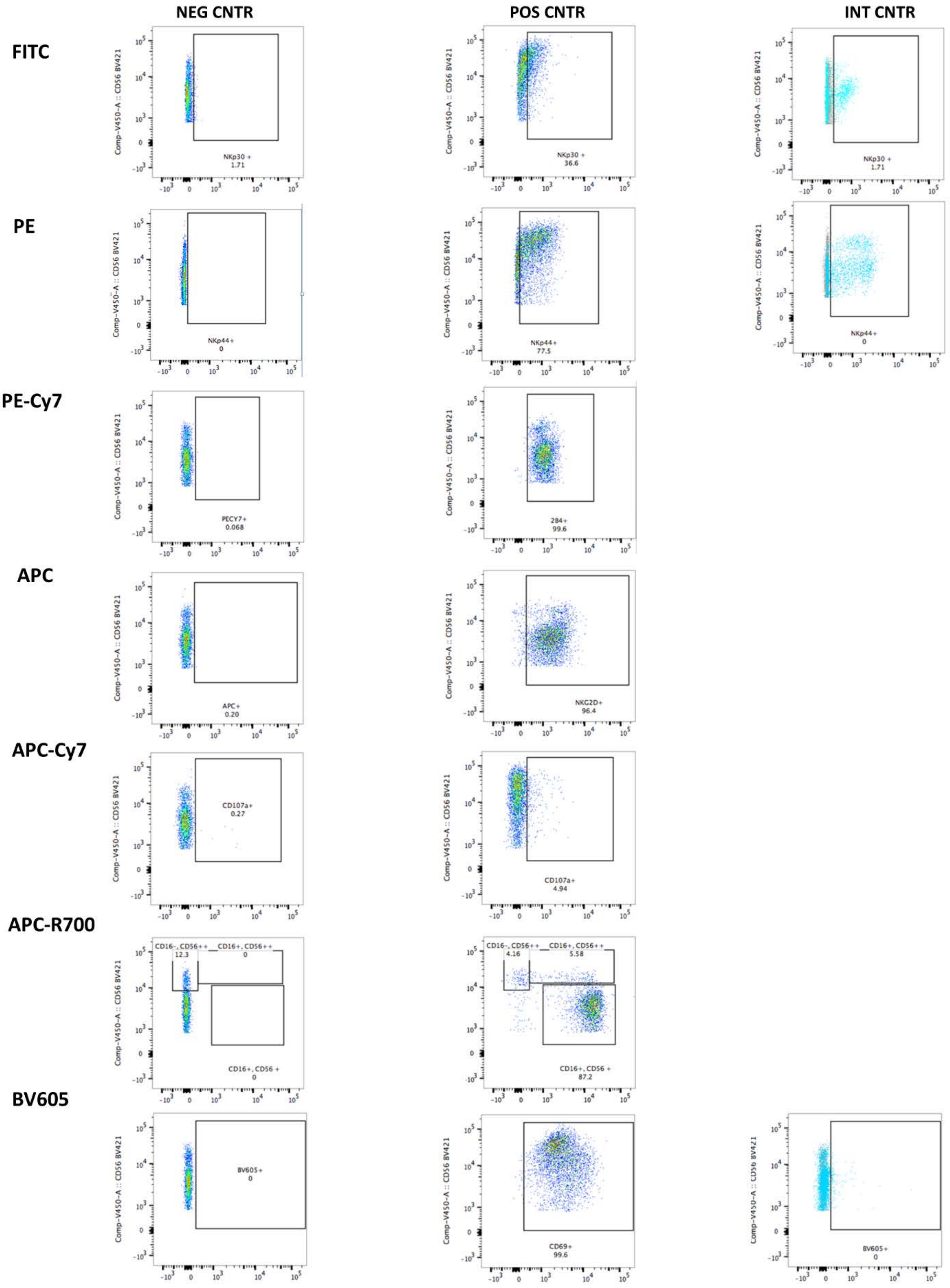

Supplemental Figure 5: Gating strategy of NK cell phenotyping markers. Gates defining the positive percentage of NK cell populations were set using negative controls (NEG CNTRL), positive controls (POS CNTRL) and internal controls (INT CNTRL).

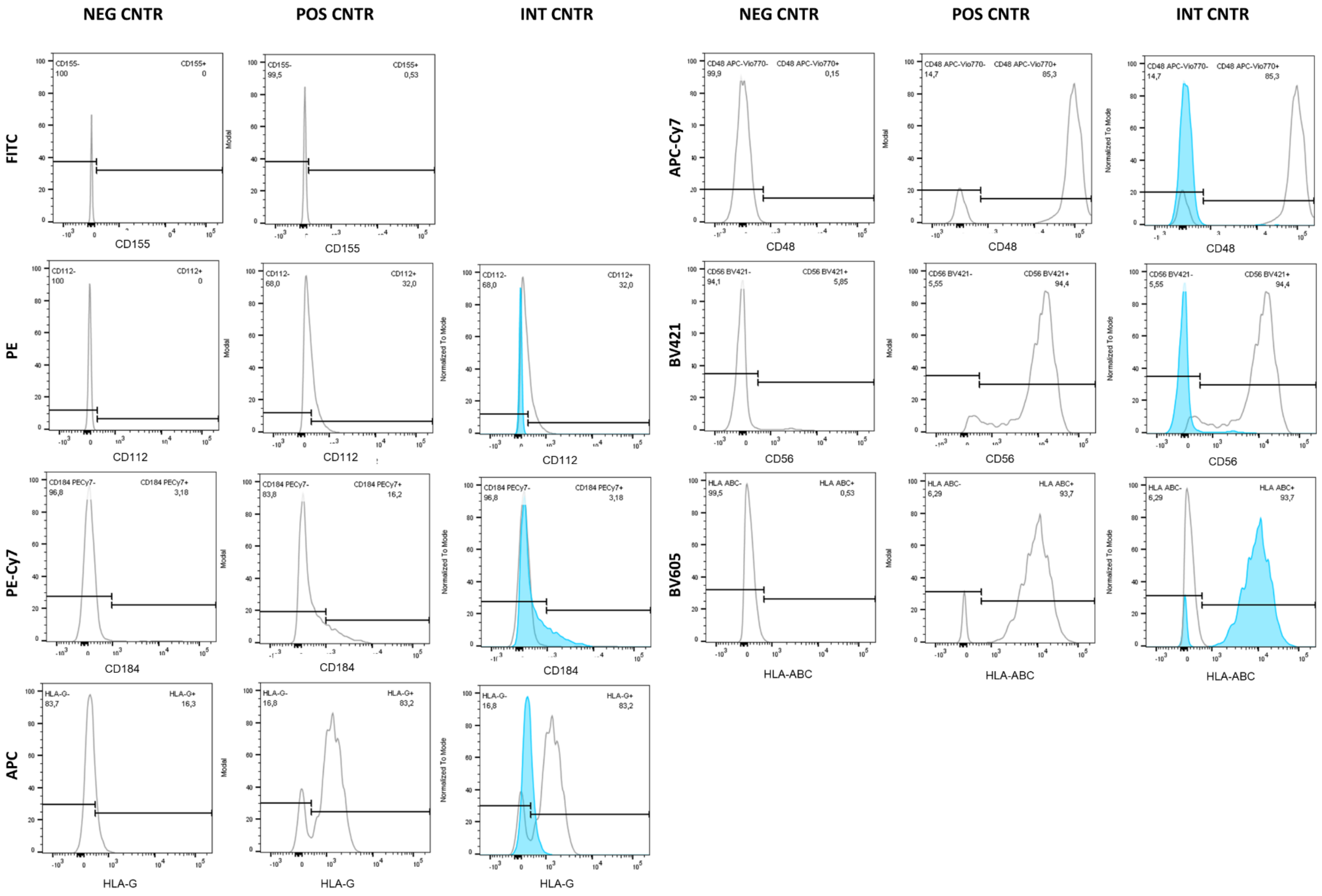

Supplemental Figure 6: Gating strategy of multiple myeloma phenotyping markers. Gates defining the positive percentage of MM cells expressing a specific surface marker were set using negative controls (NEG CNTRL), positive controls (POS CNTRL) and internal controls (INT CNTRL).

U266	OPM-2	LP-1
A02:01; 03:01	A24:02	A26:01; 30:01
B07:02; 40:01	B07:02; 15:27	B18:01; 15:27
C03:04; 07:02	C04:01; 07:02	C04:01; 07:02

Supplemental Table 1: HLA Class I genotyping of the different MM cell lines. U266, OPM-2 and LP-1 cell lines were genotyped by the German Red Cross Blood Donor Service, in order to determine the HLA Class I alleles (HLA-ABC). The three different cell lines show different sets of alleles.

	Literature	STR analysis
Amelogenin	X, Y	X, Y
CSF1PO	12, 13	12, 13
D13S317	12	12
D16S539	10	10
D5S818	11, 12	11, 12
D7S820	11, 12	11, 12
THO1	5, 7	5, 7
TPOX	8	8
vWA	17	17

Supplemental Table 2: STR (short tandem repeats) analysis of the Multiple Myeloma cell line U266.
